# Neighborhood ‘Disamenities’: local barriers and cognitive function among Black and white aging adults

**DOI:** 10.1186/s12889-023-15026-x

**Published:** 2023-01-30

**Authors:** Wenshan Yu, Michael Esposito, Mao Li, Philippa Clarke, Suzanne Judd, Jessica Finlay

**Affiliations:** 1 Program in Survey and Data Science, Institute for Social Research, University of Michigan, 426 Thompson Street, Ann Arbor, MI 48104 USA; 2 Social Environment and Health, Survey Research Center, Institute for Social Research, University of Michigan 426 Thompson Street, Ann Arbor, MI United States 48104; 3 Department of Sociology, Washington University in St. Louis, St. Louis, MO 63130 USA; 4 Center for Social Epidemiology and Population Health, Department of Epidemiology, School of Public Health, University of Michigan, 1415 Washington Heights, Ann Arbor, MI 48109 USA; 5 School of Public Health, University of Alabama at Birmingham, 1665 University Blvd, Birmingham, AL 35233 USA

**Keywords:** Cognitive function, Neighborhood, Urban health, Aging

## Abstract

**Background:**

This study examined the association between cognitive function and three neighborhood ‘disamenities’ that may pose local barriers to utilizing community resources and increase risk for cognitive decline.

**Method:**

Using national data from 21,165 urban- and suburban-dwelling Black and white adults (mean age: 67 years) in the Reasons for Geographic and Racial Differences in Stroke (REGARDS) Study, we assessed global cognitive function through a factor score of five cognitive screening tests. General Additive Mixed Models (GAMM) tested whether residing in areas with more polluting sites, highways, and limited walkability was associated with worse cognitive function.

**Results:**

Limited walkability and the presence of polluting sites had a significant negative association with cognitive function after controlling for individual and neighborhood factors.

**Conclusion:**

Neighborhood disamenities may be linked to cognitive function among aging residents. Identifying neighborhood factors that pose barriers to accessing community resources may inform upstream policy applications to reduce risk for cognitive decline.

**Supplementary Information:**

The online version contains supplementary material available at 10.1186/s12889-023-15026-x.

## Background

Neighborhood environments shape health behaviors and lifestyles. People exercise, socialize, and live a large proportion of daily life within their neighborhoods. Neighborhoods are associated with multiple health and wellbeing outcomes, such as obesity, depression, and diabetes [1]. The relationship between neighborhoods and health may be especially pronounced for aging populations since they typically spend more time in their homes and immediate surroundings given physiological and social factors associated with later life, including comorbid health conditions, mobility limitations, and retirement [2].

Compared to other health outcomes, research on the relationship between neighborhood environments and cognitive outcomes is relatively scarce, especially among racially and geographically diverse older adults [3, 4]. Yet neighborhoods may play an important role in cognitive health by exposing residents to environmental toxins such as air pollution and noise [5] and affecting individuals’ lifestyle behaviors [6]. Evidence is needed to inform the emerging ecological model of cognitive function [7] to critically investigate which environments may pose greater risk for cognitive impairment and dementia. There are an estimated 6.5 million Americans aged 65 and older living with Alzheimer’s dementia [8]. The global number of people with dementia is projected to reach 152 million in 2050 [9]. Identifying upstream neighborhood resources that are potential sources of cognitive reserve [10, 11] has important policy applications to help delay or prevent cognitive decline.

Previous studies have examined how neighborhood amenities are associated with later-life cognitive function. Findings indicate that having a higher proportion of recreational sites [12], community centers [13], access to coffee shops and fast-food restaurants [14], senior centers, civic/social organizations [15], green space [16], parks [4], and museums and galleries [17] in one’s neighborhood is positively associated with cognitive function. Scholars have posited that neighborhoods with these attributes facilitate physical activity [18], social interaction [19], and intellectual stimulation [17], all of which are strongly linked to cognitive function [20–23]. While existing evidence focuses on the neighborhood attributes that are positively associated with later-life cognitive outcomes, little is known about the potential role of negative neighborhood factors and lifestyle barriers to cognitive function. An exception is air pollution, which is well-studied for associations with cognitive outcomes [5, 24, 25]. Evidence from animal experiments suggests airborne particulate pollutants expedite neurodegenerative processes [24]. A systematic review of longitudinal studies found that exposure to air pollutants (Particulate Matter 2.5[*PM*_2.5_], Nitrogen Dioxide [*NO*_2_], and carbon monoxide) was associated with increased dementia risk [25]. In addition, traffic proximity, which can produce air and noise pollution, was linked to cognitive impairment in multiple recent studies [24, 26].

Weiss et al. [27] demonstrated that negative characteristics of a neighborhood environment (termed “neighborhood disamenities”) might affect access to local health-promoting facilities such as parks. Their findings emphasized that proximity to health amenities does not necessarily imply access, since hazards and disincentives such as crime, lack of pedestrian safety, and noxious land uses might dissuade people from using parks or recreational facilities [27]. Motivated by this observation, our study investigated whether neighborhood disamenities are negatively associated with cognitive function in a nationwide study of aging Americans.

We considered limited walkability, the presence of polluting sites, and proportion of highways as neighborhood disamenities. These measures reflect barriers that residents face to be mobile and engage in activities that promote physical, mental, and social health, which, as discussed previously, are linked to improved cognitive aging outcomes. Higher neighborhood walkability has been found to be associated with physical activity [18, 28], fewer depressive symptoms among older men [29], and less isolation [30]. Polluting sites and highways may serve as physical barriers to community resources, in addition to being sources of pollution that can pose risks for cognitive decline [24, 25]. Based on these findings, we hypothesized that neighborhood disamenities are negatively associated with cognitive function and tested this hypothesis in a nationwide study of aging Americans.

## Methods

### Design

We examined whether and how neighborhood disamenities were associated with cognitive function in a national cohort of older Americans in the REasons for Geographic And Racial Differences in Stroke (REGARDS) Study. We geocoded the REGARDS participants’ residential addresses and merged their cohort survey with neighborhood community profiles pulled from multiple contextual data sources.

### Data

The REGARDS Study is an ongoing cohort study examining regional and racial differences in stroke and cognitive function. It covers the 48 continental United States and oversamples in the Stroke Belt, a region of high stroke mortality in the Southeastern US (North Carolina, South Carolina, Georgia, Alabama, Mississippi, Tennessee, Arkansas, and Louisiana) [31, 32]. The baseline data was collected from January 2003 to October 2007 from 30,239 Black and white participants in the continental United States [31]. A cognitive battery was first implemented in 2006 and followed up at two-year intervals. Participants’ residential addresses were recorded during the baseline and follow-up periods. The University of Alabama at Birmingham Institutional Review Board annually reviews and approves ongoing study procedures, and all participants provided informed consent.

The data for this analysis included urban and suburban dwelling participants (defined by rural-urban commuting area codes) [33] who participated in at least one cognitive assessment between 2006 and 2017 and had a geocoded residential location. The final sample included 21,165 participants from 12,675 unique census tracts, contributing to a total of 73,263 records. The number of cognitive tests per participant ranged from 1 to 7 (median: 3).

### Outcome: cognitive function

Cognitive assessments in REGARDS included: 1) the Consortium to Establish a Registry for Alzheimer’s Disease Word List Learning (WLL), which assesses verbal learning [34]; 2) Word List Delay Recall (WLD), assessing verbal memory [34]; 3) Animal Fluency Test (AFT), which evaluates semantic memory and executive function; 4) Letter Fluency Test (LF), which tests language and executive function; and 5) a subset of the Montreal Cognitive Assessment (MoCA) to assess verbal memory and orientation. We developed a composite measure of cognitive function through a confirmatory factor analysis (CFA) of all five above-mentioned cognitive assessments. The CFA model fit the data well (Root Mean Square Error of Approximation = 0.013; Comparative Fit Index = 0.999). Further details on the cognitive tests and the factor structure of the model can be found in the Supplementary Information (Table S1).

### Exposure: neighborhood Disamenities

The first measure, lack of walkability, was adapted from the Environmental Protection Agency (EPA) Walkability index. This index consisted of block groups ranked according to the relative walkability of the built environment [35]. The index was a continuous variable, ranging from 1 to 19.833 and following an approximately normal distribution. The EPA calculated the index based on the equal weighting of the following three factors: 1) intersection density, 2) mix of employment and household types, and 3) percent of workers who carpool. We reverse-coded the variable to reflect the level of limited walkability (i.e., the larger the value, the less walkable the census block group). The index was constructed for every Census 2010 block group.

The second measure was the presence of polluting sites. The measure was a binary variable, with 0 indicating no polluting sites within a participant’s census tract boundary plus a half-mile buffer, and 1 indicating the presence of at least one polluting site in the area. The measure was derived from the EPA’s Toxics Release Inventory (TRI) Program [36]. Facilities that reported to TRI were typically larger facilities involved in manufacturing, metal mining, electric power generation, chemical manufacturing, and hazardous waste treatment. Based on TIGER/Line Shapefiles of the 2010 Census tracts from the US Census Bureau [37], we matched the location of polluting sites to census tracts. We incorporated the half-mile buffer to account for edge effects (e.g., if a participant lived near the edge of a census tract whose activities were constrained by a nearby polluting site just outside the census tract boundary). This measure was time-varying, as the location of polluting sites was updated yearly by TRI. If a participant moved during the study period (2006–2017), we updated their polluting sites measure accordingly.

The third measure was the amount of highways, derived through the proportion of the street length of primary and secondary roads among all street types per census tract [38]. Primary roads are generally divided interstate highways (distinguished by the presence of interchanges). Secondary roads are main arteries, usually in the US Highway, State Highway, or County Highway systems. This measure was created based on the 2010 US Census geography [37].

### Covariates

To explore the net effects of the three neighborhood features, we controlled for individual and census tract-level demographic and socioeconomic status (SES) variables. Individual-level covariates included participants’ age at baseline, number of years after the first assessment, education level, and race. Tract-level covariates included Rural-Urban Commuting Area (RUCA) type [33], the proportion of residents who were Non-Hispanic Black, the proportion of the population living below the poverty line, the proportion of housing units that were owner-occupied [39], and the average population size per square mile in a tract [40]. Since population density followed a right-skewed distribution, we took the cubic root of the original value to attenuate the effects of extreme values. In addition, we controlled for potential practice effects by including a variable indicating whether it was the participant’s first cognitive test [41].

### Analysis

This study assessed the association between the time-varying cognitive function scores (2006–2017) and three neighborhood disamenity measures: one time-varying (polluting sites: 2006–2017) and the other two time-invariant (lack of walkability and highways were measured only in 2010). Pooling data across waves, we used a multilevel linear regression model (Model 1) to regress cognitive function on individual and neighborhood socio-demographic variables, population density, and whether it was the first cognitive test. By pooling multiple waves of data, we can make inferences about the cross-sectional association between cognitive function and neighborhood disamenities averaged across multiple time points (2006–2017). We specified random effects at individual and tract levels, to account for clustering of observations within individuals and census tracts.

Model 2 added the three neighborhood disamenity measures. Because little was known about the relationships between the neighborhood disamenity measures and cognitive function, we applied Generalized Additive Mixed Models (GAMM) [42] to describe their associations. Compared to linear models, GAMM can detect and describe nonlinear relationships between predictors and outcome variables. We placed penalized thin plate regression splines on the lack of walkability index and the proportion of highways. We added the binary variable whether having polluting sites as a covariate and reported whether it was statistically significant based on the Wald-type T tests. We tested whether the smooth terms were statistically significant using F tests and reported the statistics and *P* values [43]. We used the gamm4 function from the gamm4 package [42] in R [44] to implement Model 1 and Model 2.

Because the associations between cognitive function, lack of walkability, and the proportion of highways cannot be observed from the model output directly, we calculated the predicted values of cognitive scores with 95% confidence intervals for the measures based on Model 2.

## Results

Table 1 presents the descriptive characteristics of the REGARDS analytic sample. The mean age of the participants was approximately 66.97 years (standard deviation [SD]: 8.83). 40% were non-Hispanic Black, 56% female, and 68% had at least some college education. The average cognitive function factor score was 0.01 (SD: 2.36). In our analytical data, the average lack of walkability index was approximately 9.05 (SD: 3.88), the average proportion of highways was 0.10 (SD: 0.08), and 17% lived in neighborhoods with polluting sites present. On average, each participant contributed almost three records (2.66, SD: 1.52) to the data.

**Table 1:** Descriptive statistics of the analytical sample (*n* = 21,165)

Variable	Mean/proportion	Standard deviation
Cognitive test score	0.01	2.36
Age (at baseline test)	66.97	8.83
Black	0.40	–
Female	0.56	–
Education: less than high school	0.09	–
Education: high school	0.24	–
Education: some college	0.27	–
Education: college or higher	0.41	–
Metropolitan type: core	0.88	–
Tract: proportion Black	0.42	0.35
Tract: proportion earning below poverty line	0.19	0.13
Tract: proportion of housing owner occupied	0.63	0.21
Lack of walkability	9.05	3.88
Proportion of highways	0.10	0.08
Having polluting sites in the neighborhood	0.17	–
Years since baseline test	3.51	3.20
Population density (cubic root)	13.43	6.27
Number of cognitive tests	2.66	1.52

*Notes:* respondents contributed 73,263 observations to the sample and were clustered within 12,675 unique census tracts. Presence of polluting sites and years since baseline test are time-varying covariates. Their means were calculated across all observations

Table 2 presents the parameter estimates and standard errors for the two models regressing cognitive function on individual and neighborhood features. Results from Model 1 showed that lower cognitive function scores were significantly associated with older age, Black race, male, lower educational attainment, poorer neighborhood SES, lower population density, and first time taking cognitive tests.

**Table 2:** Generalized additive mixed models of cognitive function and neighborhood disamenities

	Model 1		Model 2	
**Fixed effects**				
Parameters	Coefficients (SE)		Coefficients (SE)	
Intercept	6.48***(0.12)		6.60***(0.12)	
Baseline age	-0.09***(0.00)		-0.09***(0.00)	
Years from baseline	-0.08***(0.00)		-0.08***(0.00)	
White (ref. Black)	0.94***(0.03)		0.95***(0.03)	
Male (ref. Female)	-0.34***(0.02)		-0.34***(0.02)	
Education: college degree (ref.)	–		–	
Education: some college	-0.69***(0.03)		-0.69***(0.03)	
Education: high school	-1.19***(0.03)		-1.19***(0.03)	
Education: less than high school	-1.80***(0.04)		-1.79***(0.04)	
Metro type: urban core (ref.)	–		–	
Metro type: non-core	-0.02(0.04)		-0.01(0.04)	
Tract: proportion owner occupied housing	0.21**(0.07)		0.19**(0.07)	
Tract: proportion Black	-0.16***(0.04)		-0.15***(0.04)	
Tract: proportion below poverty line	-0.40***(0.11)		-0.40***(0.11)	
Population density (cubic root)	0.02***(0.00)		0.01**(0.00)	
Whether first time cognitive tests	-0.09***(0.02)		-0.09***(0.02)	
Not having polluting sites in the neighborhood (ref.)	–		–	
Having polluting sites in the neighborhood	–		-0.07**(0.03)	
**Smooth effects**				
	EDF	F statistic	EDF	F statistic
Smooth term for lack of walkability	–	–	2.70	6.35***
Smooth term for proportion of highways	–	–	1.00	3.36.
**Random effects**				
	SD	LR test statistic	SD	LR test statistic
Person-specific intercepts	1.26	11,993.80***	1.26	11,990.00***
Person-specific slopes for years since the baseline	0.13	1160.00***	0.13	1162.50***
Tract-specific intercepts	0.26	23.60***	0.25	37.00***
	AIC = 286,891.80		AIC = 286,878.30	

*Notes:* “SE” stands for “standard error.” Ref. indicates “reference category.” EDF means “empirical degrees of freedom.” SD indicates “standard deviation.” LR refers to “likelihood ratio.” *** *p* < 0.001, ** *p* < 0.01, * *p* < 0.05,. *p* < 0.1. The F test statistics were used to test whether the smooth terms were statistically significant [43] and produced from the gamm4 package in R [45]. The likelihood ratio test statistics were calculated by subtracting the − 2 Restricted Maximum Likelihood (REML) log-likelihood of the model with both person-specific intercepts, person-specific slopes for years since the baseline, and tract-specific intercepts from a reduced version of the model excluding the random effects [46]

Model 2, displayed in the second column of Table 2, presents the results after adding the three neighborhood disamenity measures to the model. Residents living in neighborhoods with polluting sites had a 0.07 (SE: 0.03, *p* < 0.01) lower cognitive score than residents living in neighborhoods with no polluting sites. We cannot directly observe the relationships between lack of walkability, the proportion of highways, and cognitive function from Table 2. Yet, from the F test statistics, we found strong evidence that there was a non-zero association between the lack of walkability and cognition (*p* < 0.001). In addition, we evaluated whether interactions between race and disamenity measures should be included in the model and did not find evidence suggesting that the associations differed by race.

**Fig. 1:**
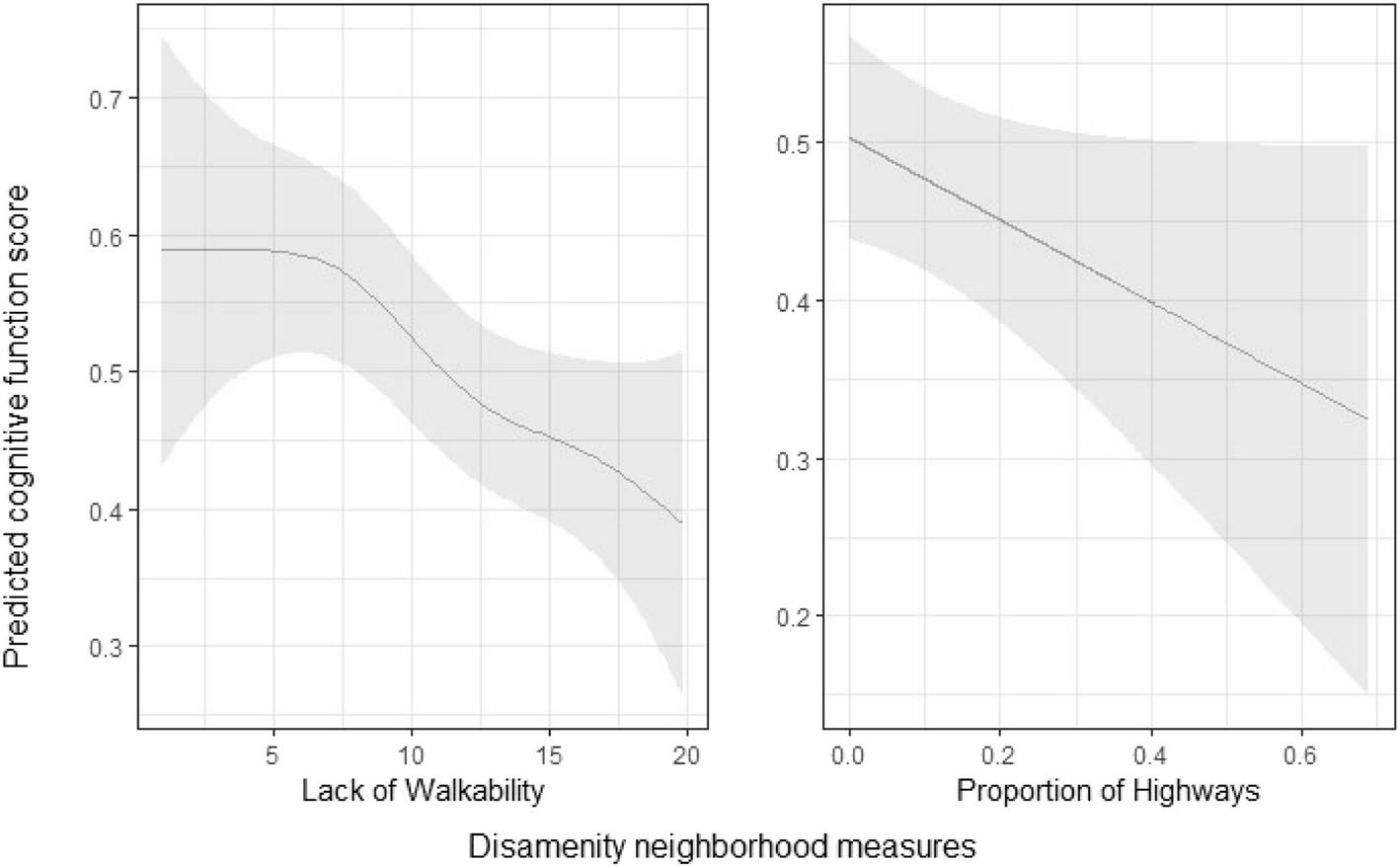
Predicted cognitive test scores across a range of neighborhood disamenity measures. *Notes:* The grey regions reflect 95% uncertainty intervals. We visualized the associations between cognitive function, the lack of walkability, and the proportion of highways because it is not possible to directly observe the associations from the output from the generalized additive model

Figure 1 visualizes the predicted relationship between cognitive function, lack of walkability, and proportion of highways based on Model 2. For residents living in neighborhoods that are the least walkable, the predicted cognitive function score was approximately 0.20 lower than residents living in neighborhoods that are more walkable (at the lowest end of the index, 0.59–0.39). This effect is comparable to 2.2 years differences in the baseline age of participants, as the estimated coefficient of baseline age is -0.09 in Model 2.

In the right panel, there is a negative linear association between the predicted cognitive scores and the proportion of highways. This is consistent with the results reported in Table 2 (Model 2). Across the whole range of the proportion of highways in census tracts (0 to approximately 0.7), the predicted cognitive function scores were 0.18 units lower (0.50–0.32), similar to 2 years differences in the baseline age of participants.

## Discussion

The neighborhood disamenity measures we examined were based on the premise that they operate as barriers for residents to access cognitively-supportive neighborhood amenities. For example, large factories and highways either deter walking or produce unpleasant odors or noise, thus preventing residents from using nearby and outdoor amenities. However, we recognize that the presence of pollution sites and the proportion of highways are also linked with cognitive health through vascular mechanisms [5, 25]. Literature suggests that exposure to *PM*_2.5_, *NO*_2_, and carbon monoxide is associated with increased dementia risk [25]. Moreover, emerging evidence shows that living in proximity to major roads is adversely correlated with cognitive health partly due to exposure to air pollutants [24, 26]. However, air pollutants did not fully explain the association between traffic proximity and cognitive impairment [24]. Our work validates and extends this area of cognitive health research by considering both biological and lifestyle perspectives.

The results of this study also provide greater insights into the limited research on walkability and cognition among older adults. Cross-sectional data in England suggested that living in areas with the highest quintile of land use mix was found to be significantly associated with reduced odds of dementia [47], and neighborhood walkability was linked to better cognition-related neuroimaging outcomes [48]. However, another study using cross-sectional data in six US regions found that greater social destination density, walking destination density, and intersection density were associated with worse cognitive performance, especially among non-white participants [12]. Our results indicated that a lack of walkability was associated with worse cognitive performance among aging populations, thus adding to the evidence that living in more walkable neighborhoods may enhance cognitive reserve.

This study extends the current line of research on neighborhood contexts and cognition by investigating how neighborhood disamenities may be associated with cognitive outcomes. Existing literature tends to focus on how proximity to or density of built and social infrastructure is linked to higher cognitive outcomes by providing space for residents to ‘get out the door’, exercise, and socialize [6, 12, 13, 15]. However, the availability of neighborhood amenities may not necessarily confer benefits to cognitive function, since access to the amenities can be affected by other neighborhood contextual factors [27] in addition to personal preference and structural societal inequalities [17]. As the first exploratory study to investigate the association between neighborhood disamenities and cognition, we consider our results as hypothesis-generating and hope to motivate more scholarly attention to this line of research.

There are important study limitations to note. First, we did not have cognitive domain-specific a priori hypotheses to test; therefore, we cannot determine if the neighborhood disamenities were linked to specific cognitive abilities. Second, we assumed the measures of limited walkability and highways were constant during the study period and used 2010 data to approximate the exposure participants received. These neighborhood features, such as highway infrastructure, are generally fairly stable across time but may miss some urban redevelopment and construction. Third, we used census tracts and block groups as proxies for neighborhoods. While this approach can be suboptimal because neighborhood boundary and size can be heterogeneous, it is a common approach when no exact measure of neighborhoods is available and in large study samples [3, 12, 13, 27]. Fourth, this study did not control for some individual covariates such as depression, wealth, and marital status because they were not updated regularly or directly measured in our data. Last but not least, REGARDS is not a nationally representative sample, so findings in this study may not be generalizable to the older population in the US. However, it does include a large geographically and racially diverse cohort of Black and white aging Americans.

## Conclusion

This study is one of the first to investigate the relationship between neighborhood disamenities [27] and later-life cognitive function in a large, racially, and geographically diverse cohort of aging adults. We measured the neighborhood disamenity concept (i.e., hazards and barriers to healthy behaviors and service access) with three local area variables. Findings indicate that a lack of walkability and the presence of local polluting sites were negatively associated with cognitive function.

Our study supplemented existing knowledge about how neighborhood resources are linked to dementia risk by investigating three disamenity measures. The results call for further research on potential neighborhood disamenities and the nuanced interplay of neighborhood contextual features (i.e., how disamenities modify the associations between amenities and cognitive function). In addition to policy efforts investing in neighborhood and community resources (e.g., parks, senior centers, arts and culture amenities), our study expands the framing of policies to evaluate current hazards and barriers to accessing local sites that are potential sources of cognitive reserve. While racial/ethnic minorities and lower-income populations might have higher spatial access to parks and other amenities, these populations disproportionately experience worse health outcomes [27, 49], including a higher risk for cognitive decline and Alzheimer’s Disease and Related Dementias (ADRD) [8]. This may be in part due to the unequal placement of hazardous and toxic land uses, lack of investment in walkability, and unsupportive urban design across neighborhoods. Therefore, tackling neighborhood disamenities may be critical to support healthier lifestyles and cognitive outcomes among diverse aging Americans.

## Supplementary Information

**Additional file 1.** Table S1. Cognitive battery tests contributing to the global cognitive function factor score

## Data Availability

Datasets for polluting sites can be found at https://www.openicpsr.org/openicpsr/project/159961/version/V1/view. Datasets for highways are available at https://www.openicpsr.org/openicpsr/project/159902/version/V1/view. Walkability datasets can be found at https://www.epa.gov/smartgrowth/smart-location-mapping#walkability. REGARDS datasets analyzed during the current study are not publicly available due it contains sensitive personal and health information but may be available if apply to REGARDS study executive committee for data access (check https://www.uab.edu/soph/regardsstudy/researchers for procedures).

## Abbreviations

REGARDS: Reasons for Geographic and Racial Differences in Stroke
GAMM: Generalized Additive Mixed Model
*PM*_2.5_: Particulate Matter 2.5
*NO*_2_: Nitrogen Dioxide
WLL: Word List Learning
WLD: Word List Delay
AFT: Animal Fluency Test
LF: Letter Fluency
MoCA: Montreal Cognitive Assessment
CFA: Confirmatory Factor Analysis
EPA: Environmental Protection Agency
TRI: Toxics Release Inventory
SES: Socioeconomic Status
RUCA: Rural-Urban Commuting Area
SD: Standard Deviation
SE: Standard Error
Ref: Reference category
EDF: Empirical Degrees of Freedom
LR: Likelihood Ratio
REML: Restricted Maximum Likelihood
ADRD: Alzheimer’s Disease and Related Dementias
