# Supplementary material for: Neighborhood ‘Disamenities’: local barriers and cognitive function among Black and white aging adults
Source: BMC Public Health. 2023 Jan 30;23:197. doi: 10.1186/s12889-023-15026-x (PMC9885664; doi:10.1186/s12889-023-15026-x)
Supplement: Supplementary file 1 — Additional file 1. Table S1. Cognitive battery tests contributing to the global cognitive function factor score [file 12889_2023_15026_MOESM1_ESM.docx]

**Supplementary Information**

**Table S1.** Cognitive battery tests contributing to the global cognitive function factor score

| **Cognitive Test** | **Score Range** | **Cognitive Domain** |
| --- | --- | --- |
| World List Learning (WLL) | 0-30 | Verbal learning |
| Word List Delayed (WLD) | 0-10 | Verbal memory |
| Animal Fluency Test (AFT) | Number of unique animals named in 1 minute | Language and executive function |
| Letter Fluency Test (LF) | Number of unique words beginning with the letter “F” named in 1 minute | Language and executive function |
| Montreal Cognitive Assessment (MoCA) subset ^a^ | 0-30 | Verbal memory and orientation |

*Notes:* Factor loadings ranged from 0.43 (MoCA) to 0.79 (AFT), and model fit improved when allowing for correlated error among the memory items (WLL, WLD, MoCA) (Root Mean Square Error of Approximation=0.013; Comparative Fit Index=0.999).

*^a^* MoCA subset: 5-word memory registration, 5-word delayed memory recall, 6-item orientation, 1-letter phonemic fluency.
